# A small-molecule approach to restore female sterility phenotype targeted by a homing suppression gene drive in the fruit pest *Drosophila suzukii*

**DOI:** 10.1371/journal.pgen.1011226

**Published:** 2024-04-05

**Authors:** Suhan Ma, Xuyang Ni, Shimin Chen, Xiaomu Qiao, Xuejiao Xu, Weizhe Chen, Jackson Champer, Jia Huang

**Affiliations:** 1 Ministry of Agriculture Key Laboratory of Molecular Biology of Crop Pathogens and Insects, Institute of Insect Sciences, Zhejiang University, Hangzhou, China; 2 Xianghu Laboratory, Hangzhou, China; 3 Center for Bioinformatics, School of Life Sciences, Center for Life Sciences, Peking University, Beijing, China; 4 PTN program, School of Life Sciences, Tsinghua University, Beijing, China; The University of North Carolina at Chapel Hill, UNITED STATES

## Abstract

CRISPR-based gene drives offer promising prospects for controlling disease-transmitting vectors and agricultural pests. A significant challenge for successful suppression-type drive is the rapid evolution of resistance alleles. One approach to mitigate the development of resistance involves targeting functionally constrained regions using multiple gRNAs. In this study, we constructed a 3-gRNA homing gene drive system targeting the recessive female fertility gene *Tyrosine decarboxylase 2* (*Tdc2*) in *Drosophila suzukii*, a notorious fruit pest. Our investigation revealed only a low level of homing in the germline, but feeding octopamine restored the egg-laying defects in *Tdc2* mutant females, allowing easier line maintenance than for other suppression drive targets. We tested the effectiveness of a similar system in *Drosophila melanogaster* and constructed additional split drive systems by introducing promoter-Cas9 transgenes to improve homing efficiency. Our findings show that genetic polymorphisms in wild populations may limit the spread of gene drive alleles, and the position effect profoundly influences Cas9 activity. Furthermore, this study highlights the potential of conditionally rescuing the female infertility caused by the gene drive, offering a valuable tool for the industrial-scale production of gene drive transgenic insects.

## Author summary

In the quest to control disease-transmitting vectors and agricultural pests, the revolutionary CRISPR-based gene drive emerges as a powerful tool. This genetic engineering innovation manipulates the inheritance of specific genes, ensuring their pervasive spread across generations. Here, our study introduces a CRISPR-based gene drive system designed to target female fertility gene *Tyrosine decarboxylase 2* (*Tdc2*) in *Drosophila suzukii* and demonstrated moderate gene drive efficiency. Interestingly, feeding octopamine restored the egg-laying defects in *Tdc2* mutant females, allowing easier line maintenance and large-scale production of genetically modified insects. In parallel, we extended our investigations to *Drosophila melanogaster* to further refine and characterize the drive system. These findings underscore the potential of CRISPR-based gene drives to revolutionize pest management, offering promising avenues for the development of environmentally friendly solutions in agriculture.

## Introduction

Gene drive is a novel genetic engineering tool for controlling disease transmitting vectors and agricultural pests. Specifically, CRISPR/Cas9-based gene drive has broad application prospects due to its high specificity and flexibility. It functions by biasing the inheritance of specific genes to ensure their widespread transmission across successive generations. Current CRISPR-based gene drives consist of three key components: a Cas9 endonuclease gene, one or multiple gRNAs, and a marker gene. Once the target site on a homologous chromosome is cleaved, the gene-drive element is inserted into the double-stranded break (DSB) through homology-directed repair (HDR), utilizing the drive-bearing homologous chromosome as a template for repair [1]. If this process of directional gene conversion, also known as homing, is highly efficient, the drive allele will be inherited in a manner that surpasses typical Mendelian inheritance patterns (>50%). Under ideal conditions, the gene drive elements will eventually spread throughout the population. In fact, *Anopheles* mosquitoes engineered with CRISPR-based gene-drive have achieved close to 100% inheritance of the desired allele across successive generations [2].

However, the emergence of resistance alleles through end-joining can inhibit the drive, blocking recognition by the gRNAs and potentially maintaining gene function [3–6]. It should be noted that in this research we use the term "resistance" broadly refer to alleles formed by end-joining repair, causing mutations that prevent their cleavage by the drive. In some lab experiments, resistance to CRISPR/Cas9-based homing drives arise rapidly, especially when using a single gRNA [3,4,7]. Thus, a homing suppression drive targeting a female fertility gene can slow down the development of resistance alleles because any sequence alternation, including in-frame indels, would incur substantial fitness costs [2]. Because the drive itself causes recessive female sterility, another important technical aspect of this approach is how to keep large-scale rearing for area-wide releases with a homing suppression element that would otherwise crash due to female sterility.

Intriguingly, previous studies in *Drosophila melanogaster* have unveiled the potential of a specific target gene, *tyrosine decarboxylase 2* (*Tdc2*). This gene encodes an enzyme involved in the synthesis of octopamine, a neurotransmitter that plays a vital role in various physiological processes, including egg laying [8]. A single point mutation in the active site of *Tdc2*, disrupting the production of octopamine in the nervous system, results in female sterility due to impaired oviductal contractions and egg retention in homozygous females, while heterozygotes are normal [9]. More importantly, this characteristic can be restored through octopamine feeding, indicating that *Tdc2* is a potential target for gene drives and enabling conditional rescue of the homozygous drive phenotype [9].

*Drosophila suzukii*, also known as spotted wing drosophila (SWD), is a major agricultural pest. Female SWD have specialized serrated ovipositors, which can lay eggs directly into ripe fruits with soft skins such as strawberries and grapes. The sterile insect technique (SIT) has been successfully employed for the widespread suppression of pests, including SWD, by releasing a substantial excess of sterile males in a repeated manner at a ratio of at least 10:1 [10,11]. Due to the high costs of implementing SIT over large areas, genetic suppression through gene drive technology may be a more favorable option for controlling SWD populations. Targeting the female-specific exon of the *dsx* gene, a recently created CRISPR/Cas9-based split gene drive (sGD) system in SWD showed 94–99% transmission rates with a line that expressed Cas9 with two nuclear localization sequences from the *D*. *suzukii nanos* promoter. It was effective at population suppression in mathematical simulations with continuous releases of more modest size (1:4) [12].

This study reports the development of a novel CRISPR-based homing gene drive system targeting the *Tdc2* gene with the previously untested *vasa* promoter in SWD. We demonstrated conditional rescue of the *Tdc2* mutant phenotype by feeding octopamine and showed moderate levels of gene drive efficiency that specifically target the *Tdc2* locus. We also conducted additional experiments in *D*. *melanogaster* to further characterize this drive system. These findings suggest potential applications of *Tdc2*-targeting gene drives for SWD population management, paving the way for more effective and sustainable pest control strategies.

## Results

### A homing gene drive system targeting *Tdc2* in SWD

**Fig 1:**
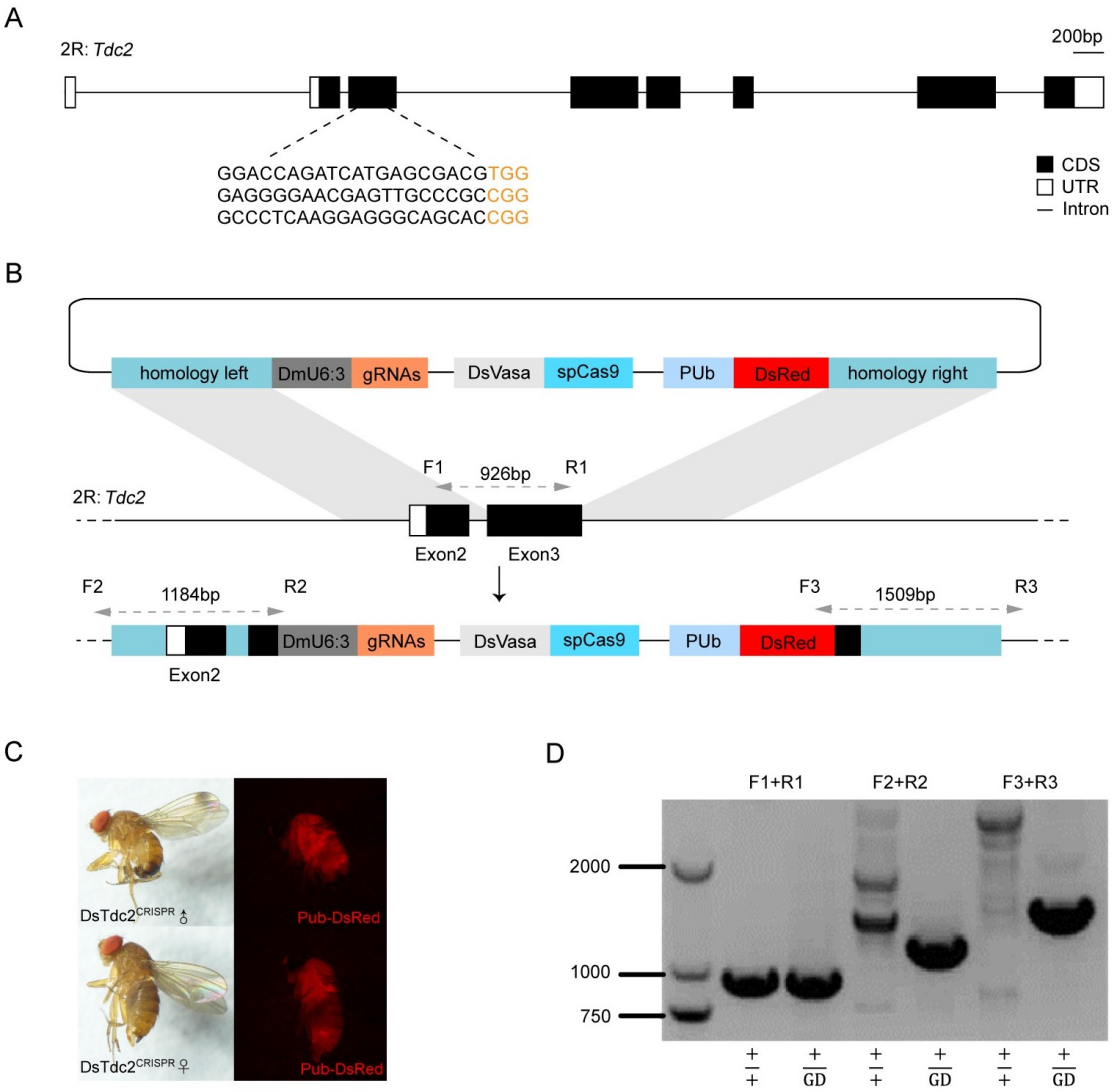
Establishment of a homing gene drive system targeting female essential gene *Tyrosine decarboxylase 2* (*DsTdc2*) in SWD. (A) Gene structure of *DsTdc2* and three gRNA sequences. (B) Schematic representation of the HDR knock-in construct of gene drive donor plasmid specifically recognizing *DsTdc2* gene and the corresponding target locus. Homology arms used to act HDR are on both sides of the U6-gRNA, *vasa*-Cas9 and PUb-DsRed. U6-gRNA and *vasa*-Cas9 are components of the fGD system and allow for a double strand break at target site. PUb-DsRed is a marker constructed to score the transgene flies. (C) Natural and red fluorescence images of male and female DsTdc2^CRISPR^. As the marker indicated, red fluorescence was observed all over the mutants under excitation light of 510 nm-560 nm. (D) Molecular confirmation of the correct integration of the HDR-mediated event to generate gene drive SWD. Primer pairs used in diagnostic PCR are shown in (B). The chromosome carrying the gene drive was named as GD.

We first analyzed the genomic structure of SWD *Tdc2* locus and selected three gRNAs with spacing between them ranging from 50–150 bp to decrease functional resistance generation and possibly improve drive efficiency [13–15] (Fig 1A). Next, we identified and synthesized the SWD *vasa* promoter for efficient germline expression of SpCas9 (S1A Fig). Homing-based suppression requires high rates of homing and low maternal carryover cleavage to prevent fitness costs and the appearance of a high frequency of NHEJ resistant alleles [3]. While the *vasa* promoter is known to have high levels of somatic expression in *D*. *melanogaster* and mosquitoes, it has not been tested in SWD. According to the *D*. *melanogaster vasa* promoter [16] and the sequence information in *vasa-*Cas*9* transgenic flies [17], we found that the commonly used *D*. *melanogaster vasa* promoter uses the 5’ UTR region in both the first and second exons, up to the start codon on the latter and excluding the intron. In contrast, the start codon of SWD *vasa* is on the fourth exon. Therefore, the 5´UTR region (112bp) on the first exon, 5´UTR region (19bp) on the fourth exon and 5´ upstream sequences (1292bp) before the first exon of SWD *vasa* were selected as the *vasa* promoter region (S1B Fig). Finally, a donor plasmid containing left and right homologous arms, tRNA-gRNAs driven by the *D*. *melanogaster U6*:*3* promoter, SpCas9 endonuclease with its *vasa* promoter sequence, and DsRed marker expressed through the whole body under the control of the *D*. *melanogaster PUb* promoter [18] was constructed to fulfill our gene drive system (Fig 1B).

After microinjection and primary outcross with wild-type (WT) SWD, flies that showed red fluorescence in the body were selected and named as DsTdc2^CRISPR^ (Fig 1C). PCR amplification from genomic DNA using diagnostic primers that indicate correct transgene integration in the *Tdc2* locus was performed to confirm that the gene drive element was inserted in the target locus (Fig 1D). The successful acquisition of mutants through microinjection showed that the SWD *vasa* promoter is functional in expressing Cas9, as observed from the outcomes when only the donor plasmid was injected without any additional Cas9 protein.

### The gene drive allele achieved super-Mendelian inheritance in SWD

**Fig 2:**
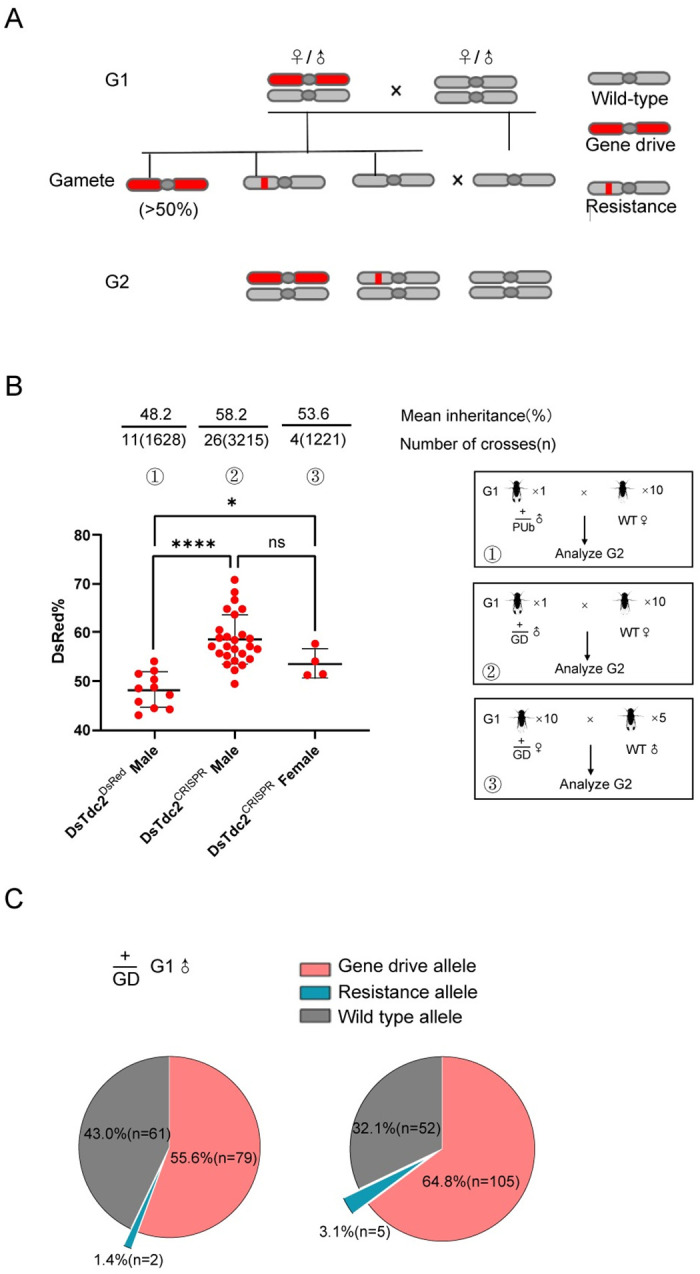
The gene drive allele integrated into *DsTdc2* achieve super-Mendelian inheritance. (A) Crossing scheme of the heterozygous DsTdc2^CRISPR^ and WT SWD. The gene drive element led to a super-Mendelian inheritance (>50%) of the construct. (B) The heterozygous male and female DsTdc2^CRISPR^ were both crossed to WT of the opposite sex and assessed for inheritance bias of the gene drive construct. Male DsTdc2^DsRed^ were also crossed to WT as a control and assessed for inheritance of the PUb-DsRed construct. Data was analyzed by unpaired two-tailed *t*-test. Stars represent statistical significance (****P< 0.0001; *P< 0.05, ns no significance) for inheritance rate. The crossing schemes of each group are shown in the black boxes. (C) The gRNA target site of the G2 generation of two male DsTdc2^CRISPR^ heterozygotes were sampled and examined. Two pie charts display the proportions of resistance alleles in the G2 generation from these two male flies. Specifically, 142 of the G2 progeny from the first male (left chart) and 162 of the progeny from the second male (right chart) were sampled and analyzed.

To test whether our gene drive system functions in SWD, we crossed DsTdc2^CRISPR^ flies with WT SWD, and the inheritance rate of the gene drive cassette was calculated by counting the number of individuals with red fluorescence among the offspring (Fig 2A). We produced another transgenic SWD without Cas9 and gRNA elements as a control (S2 Fig). The DsRed marker was inserted at the same position in the *Tdc2* locus, and such flies were named as DsTdc2^DsRed^. Males with this allele were crossed with WT female SWD (Fig 2B), and their offspring showed around Mendelian inheritance (48.2%, *P* = 0.5535, Fisher’s Exact Test).

We established 26 vials containing heterozygous DsTdc2^CRISPR^ males crossed with 10 WT female flies. These heterozygous males were regarded as the G1 generation, and their resulting offspring constituted the G2 generation. As a result, an average of 58.2% of the offspring inherited the gene drive allele, higher than Mendelian inheritance (*P*<0.0001, Fisher’s Exact Test) and also significantly higher than that in the DsTdc2^DsRed^ control group (*P*<0.0001, unpaired two-tailed *t*-test), indicating that the observed super-Mendelian inheritance in DsTdc2^CRISPR^ flies was most likely due to the gene drive activity (Fig 2B).

Unexpectedly, we found a total of five major genetic variant types for polymorphisms in the homology arms and gRNA targeting sequences in these 26 heterozygous DsTdc2^CRISPR^ males after sequencing (S3A Fig). The type (type 1) that constituted the majority of the tested G1 population (61.5%) was close to the genome-sequenced SWD strain [19], matching well with the sequences we designed for gRNAs and homology arms. The other four types of nucleotide sequences show significant differences from the reference sequence at the gRNA recognition site and the homologous arm on the right side, which may affect homing efficiency. Nevertheless, all these five genetic variants exhibited similar inheritance rates (S3B Fig).

For G1 generation heterozygous DsTdc2^CRISPR^ females, single crosses of female flies did not produce enough offspring for data analysis. Therefore, 10 heterozygous DsTdc2^CRISPR^ females were crossed with 5 WT males in each vial for four replicates. These DsTdc2^CRISPR^ females also harbored the above genetic polymorphisms, mimicking a scenario akin to a “wild population”. On average, 53.6% of the G2 progeny inherited the gene drive allele (Fig 2B).

To determine the underlying reason for the low homing rates in our gene drive system compared to those with high conversion efficiency such as 94–99% in *D*. *suzukii* [12], we isolated genomic DNA of G0, G1, and G2 DsTdc2^CRISPR^ adults to amplify the fragments surrounding the target sequence by PCR (S4A Fig). The results showed mosaic patterns in the microinjected G0 generation SWD. For G1 DsTdc2^CRISPR^, we cloned the PCR amplification products into a vector and discovered that short insertions or deletions (indels) were generated at the first and the third gRNA sites (S4B Fig). Furthermore, we sampled and examined the DsRed-negative G2 generation of two male DsTdc2^CRISPR^ heterozygotes. After cloning and sequencing the mixed PCR product, we found that most sequenced progeny inherited wild type allele (96.8% for the first male and 91.2% for the second), indicating low rates of cleavage in G1 germlines (Fig 2C). We also identified two types of NHEJ at the gRNA1 targeting site, potentially leading to either functional or nonfunctional resistance alleles (S4B Fig). The fraction of resistance alleles in the total G2 generation was 1.4% and 3% among the progeny of the two males (Fig 2C).

### An oviposition defect of DsTdc2^CRISPR^ was compensated by feeding octopamine

**Fig 3:**
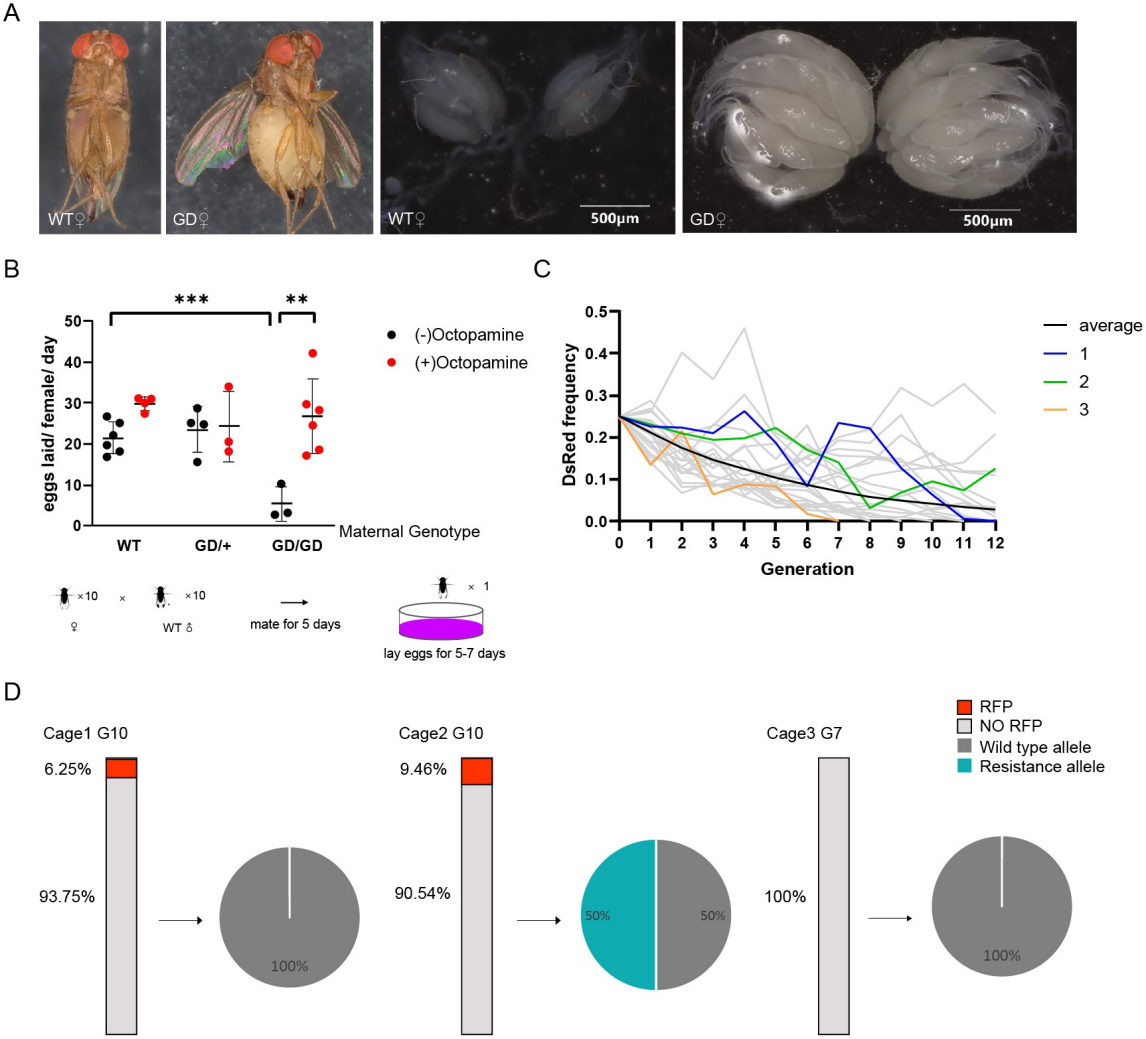
An oviposition defect of the DsTdc2^CRISPR^ can be compensated by octopamine and dynamics of the spread of the gene drive allele in cage trials. (A) Morphological analysis of WT female SWD, homozygous female DsTdc2^CRISPR^ and their ovaries. Abdominal enlargement was observed in homozygous female DsTdc2^CRISPR^ due to egg retention caused by disruption of the *Tdc2* gene. After the ovaries were dissected, it was found that the eggs of the homozygous female DsTdc2^CRISPR^ were trapped in the ovaries. (B) Reproductive phenotype of WT, heterozygous and homozygous DsTdc2^CRISPR^ with or without the addition of octopamine. Oviposition defect of homozygotes compared to WT was analyzed using unpaired two-tailed *t*-test, *n1* = 6, *n2* = 3, *P*<0.001. Octopamine compensation effect in homozygotes was analyzed using unpaired two-tailed *t*-test, *n1* = 3, *n2* = 5, *P*<0.01. (C) The genetic efficiency and model-predicted dynamics of gene drive elements in cage trials. Experimental data from three independent replicates was shown as three different colors of thick lines. 20 stimulations of the cage were shown in gray. And an “average” sample was in black. (D) GRNA cleavage situation at the 10^th^ generation of Cage 1 and 2, and the 7^th^ generation of Cage 3. Ten flies without DsRed were randomly chosen for genome typing. The rectangular squares represent the proportion of fluorescence flies in each generation. And the pie charts illustrate the editing situation at the gRNA cleavage sites in 10 randomly-chosen DsRed-negative flies separately.

In contrast to the sterile phenotype observed in *D*. *melanogaster Tdc2* mutants, we found that homozygous DsTdc2^CRISPR^ females were not completely sterile; nevertheless, they did lay fewer eggs due to the genetic disruption of *Tdc2* by the gene drive cassette. Dissection of the ovaries of 20-day-old homozygous DsTdc2^CRISPR^ also revealed enlarged ovaries and an increased number of retained embryos (20.75 embryos/ovary) compared to WT females at the same age (8.75 embryos/ovary; *P*<0.0001, unpaired two-tailed *t*-test) (Figs 3A and S5). The phenotype of the heterozygous DsTdc2^CRISPR^ females was not much different from that of the WT females, indicating that *Tdc2* is haplosufficient, requiring only a single wild-type copy for proper gene function. To test whether the feeding of octopamine can restore the female sterility phenotype, we performed an egg-laying assay. After mating 10 females of WT and DsTdc2^CRISPR^ mutants (either heterozygotes or homozygotes) with 10 WT males for 5 days, we counted the number of eggs produced by single female for 5–7 consecutive days. Because both heterozygous and homozygous DsTdc2^CRISPR^ carried red fluorescence, which were difficult to be distinguished by phenotyping, genotyping was performed after the egg-laying experiment. The results showed that homozygous DsTdc2^CRISPR^ significantly produced fewer eggs (5.40 eggs/female/day) compared to the WT females (24.46 eggs/female/day; *P*<0.001, unpaired two-tailed *t*-test). Further, there was no significant difference between the fecundity of heterozygotes (23.57 eggs/female/day) and WT (*P* = 0.2493, unpaired two-tailed *t*-test). For homozygotes, the addition of octopamine to the food compensated for the oviposition defect, restoring egg production to the WT levels (26.76 eggs/female/day; *P*<0.01, unpaired two-tailed *t*-test) (Fig 3B).

### Dynamics of the drive in cage trials

Since super-Mendelian inheritance was observed in two generations of crosses, we then tried to evaluate the effectiveness of the gene drive system in small cage populations over multiple generations. We established three replicate cages, each containing an equal number of male and female SWD. Among these flies, a quarter of both males and females (25% of the total population) were heterozygous DsTdc2^CRISPR^ (inheriting the drive from their father) while the remaining individuals were WT flies. In each generation, flies were allowed to mate and lay eggs for ~120 hours before being removed. After 16 days, all offspring were phenotyped for red fluorescence and then used to seed the next generation. This cyclic process continued for a total of 12 generations. Our results showed a steady decline in the proportion of DsRed individuals in the population (Fig 3C), the rate of which was broadly consistent with our modeling result based on drive efficiency measurements from two-generation crosses and an additional fitness cost in drive heterozygous females of approximately 47% (S1 Table).

We proceeded to examine the gRNA editing status in specific generations by selecting 10 SWD without DsRed markers from each group for genotyping. Among the tested flies, we found that all flies without markers in Cage 1 (Generation 10) and Cage 3 (Generation 7) inherited wild type allele, indicating that no cleavage had occurred in any of the alleles in these flies. However, in Cage 2 (Generation 10), 50% of the tested flies were heterozygotes with one copy of resistance allele. Among these mutants, 40% had cleavage at both gRNA1 and gRNA3 sites, and 60% had cleavage only at the gRNA3 site (Fig 3D). Sequencing chromatograms of the gene target sites in both WT flies and NHEJ mutants were analyzed and compared (S6A Fig). After cloning and sequencing of the PCR products from two flies occurring NHEJ, we identified a specific type of NHEJ at gRNA1 and gRNA3 (S6B Fig). This suggests that although gRNA-induced cleavage occurred in populations, the relatively low cleavage efficiency and homing rate resulted in the failure of spreading gene drive elements within the population.

### Gene drive systems targeting *Tdc2* in *D*. *melanogaster*

**Fig 4:**
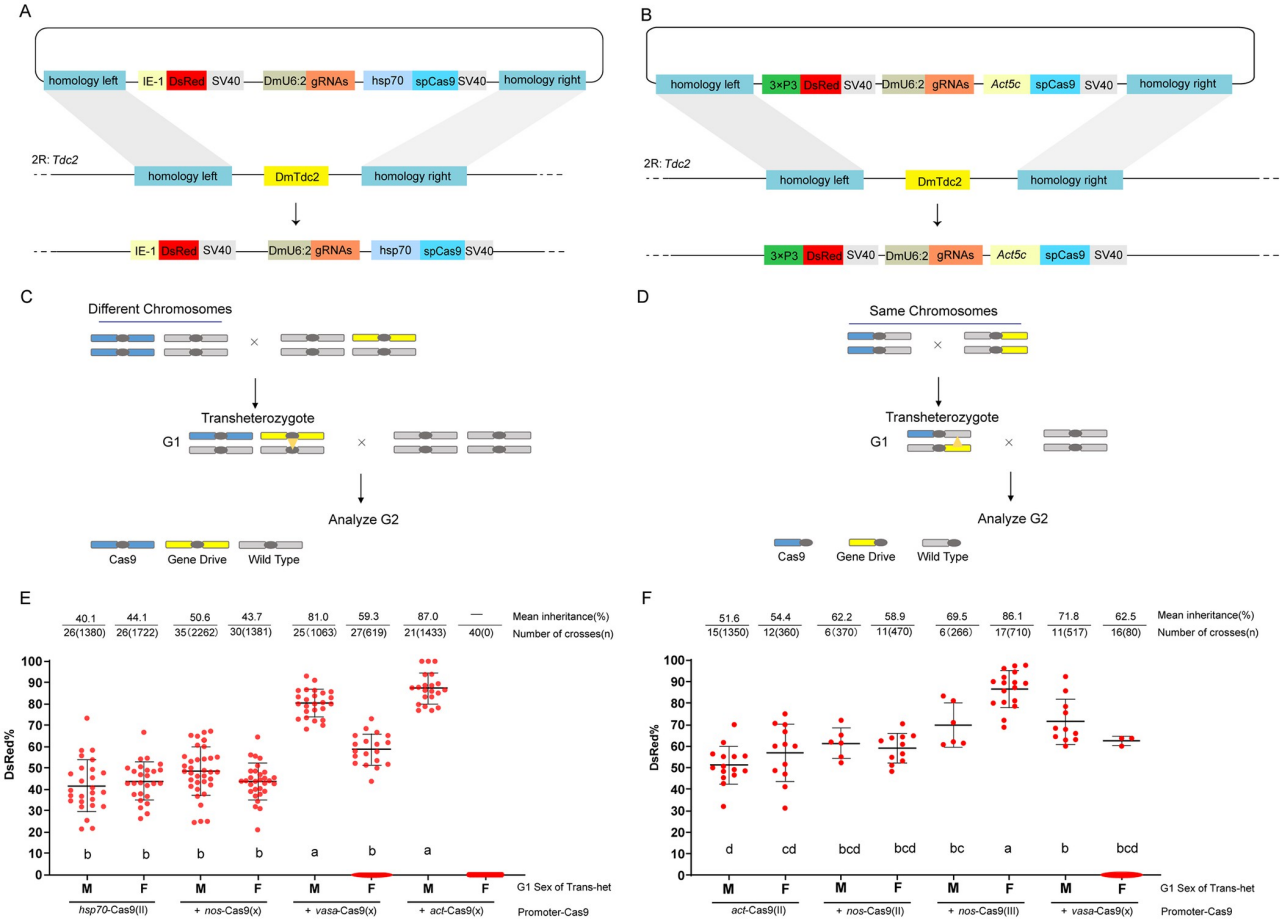
SGD systems targeting female essential gene *Tdc2* were constructed and validated in *D*. *melanogaster*. (A) and (B) Schematic representation of the HDR knock-in construct of gene drive donor plasmid specifically recognizing *DmTdc2* gene and the corresponding target locus. The plasmid contains homology arms of both ends of the cut site in order to act homology-directed repair, multi-gRNAs and Cas9 cassettes (with the ubiquitous promoter of *hsp70* or *Actin5C*) to form fGD systems, and the marker construct (IE1-DsRed for red fluorescent body or 3×P3-DsRed for red fluorescent eyes) aiming to score the transgenic flies. (C) and (D) Outline of the genetic cross schemes used to demonstrate the driving efficiency of the sGD systems. The gene drive allele and Cas9 endonuclease gene are located in either different or same chromosomes. The conversion event at the sGD locus is shown with a yellow triangle in G1 individuals. (E) and (F) For DmTdc2^hsp70^ and DmTdc2^Actin5C^, G1 flies were separately single-pair mated to *w*^*1118*^ and assessed for inheritance of the gene drive construct. The introduced Cas9 protein was marked as +. Data analyzed using one-way ANOVA (*P*<0.0001) followed by Tukey’s multiple comparison tests (letters). Note that points marked as 0 drive inheritance represent drive flies that had no offspring.

Although the constructed full gene drive (fGD) system achieved super-Mendelian inheritance in SWD, the observed low rates of cleavage (Fig 2C) may indicate low expression levels of Cas9 in the germline. At first, we hypothesized that the SWD *vasa* promoter strength might be insufficient to produce robust Cas9 expression. Therefore, we built two fGD systems in *D*. *melanogaster* and chose two efficient and ubiquitous promoters, *hsp70* and *Actin5C*, to drive expression of SpCas9 [20,21]. Donor plasmids were constructed and inserted into the same *Tdc2* locus of *D*. *melanogaster* (Fig 4A and 4B). After screening for DsRed-positive individuals containing the drive alleles (named as DmTdc2^hsp70^ and DmTdc2^Actin5C^), we first tried to determine the efficiency of these drives. Male and female heterozygous DmTdc2^hsp70^ and DmTdc2^Actin5C^ (regarded as G1 flies) were single-pair mated with *w*^*1118*^ individuals of the opposite sex. Surprisingly, our fGD system did not function as expected in both strains of flies. For DmTdc2^hsp70^, the portions of G2 offspring inheriting the gene drive cassette were 40.1% for males and 44.1% for females (Fig 4E), substantially lower than Mendelian inheritance (*P*<0.0001 for male and *P* = 0.0004 for female, Fisher’s Exact Test), perhaps due to a toxic or line effect. The inheritance rates of female (54.4%, *P* = 0.2525, Fisher’s Exact Test) and male (51.6%, *P* = 0.4150, Fisher’s Exact Test) for DmTdc2^Actin5C^ were around Mendelian inheritance (Fig 4F).

To clarify the gene editing activity mediated by *Actin5C*-Cas9, sequencing chromatograms of the *DmTdc2* gene target sites in *w*^*1118*^, heterozygous G1 DmTdc2^Actin5C^, and DsRed-negative G2 progeny were analyzed and compared (S7A Fig). We found that the G1 DmTdc2^Actin5C^ were mosaic individuals because the *Act5C* promoter is ubiquitously expressed in *D*. *melanogaster*. To quantify the proportion of individuals carrying the resistance or wild type allele in the G2 generation, we sampled the offspring of 2 male and 2 female G1 heterozygous DmTdc2^Actin5C^. The resulting proportions of individuals carrying NHEJ alleles in the G2 generation were 9% and 5% for the progeny of the males and 24% and 9% for the progeny of the females (S7B Fig). The relatively high proportions of wild type progeny (40% and 52% for the male; 35% and 20% for the female) suggested similarly low Cas9 activity as observed in DsTdc2^CRISPR^.

Since both *hsp70* and *Actin5C* promoters showed high driving efficiency in previous studies [21], we hypothesized that the *Tdc2* locus may have a negative effect on the expression of Cas9. Thus, we assessed the gene drive efficiency in pseudo-split gene drive systems (sGD), where an additional source of Cas9 was provided in *trans* from the X, II, or III chromosome and driven by various promoters (Fig 4C and 4D). For DmTdc2^hsp70^ flies, we found that the introduction of *nos*-Cas9(X) did not improve homing efficiency. However, both *vasa*-Cas9(X) and *actin5C*-Cas9(X) induced super-Mendelian inheritance of gene drive cassette, ranging from 59.3% (*P* = 0.0021, Fisher’s Exact Test) to 87.0% in terms of DsRed-positive G2 offspring. We also found that 25.9% (*vasa*-Cas9) and 100% (*actin5C*-Cas9) of G1 females were unable to lay any eggs (Fig 4E), suggesting cleavage of the *DmTdc2* gene in the somatic cells of these flies.

For DmTdc2^Actin5C^ flies, the introduction of Cas9 sources also enhanced drive efficiency, which ranged from 58.9% to 86.1% (Fig 4F). Based on the degree of improvement, we observed that the expression level of the Cas9 endonuclease followed the order of *vasa*-Cas9(X) = *nos*-Cas9(III) > *nos*-Cas9(II), consistent with previous findings [22]. In particular, 81.3% of G1 females carrying *vasa*-Cas9(X) could not lay any eggs and exhibited an enlarged abdomen phenotype due to the somatic cell activity of the *vasa* promoter (S9A Fig). In addition, we analyzed the gene editing outcomes at the gRNA cleavage sites. By isolating and sequencing PCR products from multiple DsRed-negative G2 individuals, we found that cleavage and indels mostly occurred at the gRNA2 and gRNA3 sites. In contrast, only a small proportion of individuals exhibited cleavage and indels at the gRNA1 site. Specifically, the proportion of NHEJ was 20% and 25% for two with *vasa*-Cas9(X), 12% for *nos*-Cas9(II), and 10.5% and 8% for two with *nos*-Cas9(III) (S8 and S9 Figs). These indels potentially led to the generation of functional or nonfunctional resistance alleles.

## Discussion

Our research demonstrated the robust use of the CRISPR/Cas9 genetic tool to construct fGD systems targeting the haplosufficient female fertility gene *Tdc2* in both the agricultural pest SWD and the model insect *D*. *melanogaster*. The gene drive study on SWD, an economic pest, is highly meaningful. Currently, many researchers are gradually shifting their focus from model organisms to application species and back for further exploration.

However, the fGD system implemented in SWD did not exhibit high super-mendelian homing efficiency, so it could not spread through the population in cage trials. Three factors might have contributed to this outcome. First, sequence polymorphism in gene drive modified organisms can greatly influence the efficacy of gene drive element spread in the population and thus may have negatively affected our drive [3, 6, 23]. Second, the efficiency of the Cas9 and/or gRNA expression system may have been low, perhaps due to position effects that neighboring genetic elements surrounding the transgene insertion site suppress Cas9 expression. Third, the fitness cost of the drive was moderate, too high to be overcome by the low drive efficiency. In *D*. *melanogaster*, we initially employed two Cas9 promoters that had previously shown good expression, but this did not improve homing efficiency. However, introducing Cas9 protein from another genomic source greatly increased the efficiency of the gene drive system, indicating that position effects may partially account for our SWD results.

Using gene drive to manage invasive pest SWD is a promising and innovative approach, but as a species with a high level of genetic polymorphism [24,25], such genetic background may have a significant impact on the effective spread of gene drive through populations [3,6]. In a *Medea* gene drive system of SWD [26], the genetic diversity of the population led to the emergence of antitoxin mutants in cage experiments, thus limiting the spread of the drive. The results in our study also showed that the homing rate of gene drive alleles could potentially be influenced by genetic polymorphisms in the gRNA targeting sites and homology regions.

A recent study showed high efficiency with the SWD *nanos* promoter driving expression of Cas9 [12]. In our system, the *vasa* promoter (which has usually given similar germline performance to *nanos* in *D*. *melanogaster*) element was perhaps missing too much of the middle portion of the 5’ UTR to retain normal expression levels. It is possible that including the full 5’ UTR would have increased activity. However, a single injection of donor plasmid containing gRNAs and our *vasa*-Cas9 into the embryo of SWD achieved successful genome editing, which showed, together with some drive activity, that our *vasa* promoter was functional in this system. Other homologs of promoters from *D*. *melanogaster* could potentially allow for more efficient suppression [27].

Our measured fitness cost of 47% in drive heterozygous females in the cage experiment was in line with that found in several other suppression drives [2,28,29]. This could come from drive-based disruption of wild-type alleles due to widespread somatic Cas9 expression facilitated by the *vasa* promoter. While the fecundity of heterozygous females in our individual crosses was not apparently different from wild-type, this may not be the case in population cages [29], which might be more vulnerable to changes of food condition, population density, peer competition for resources, etc. It is also possible that the observed fitness costs manifest in other ways, such as affecting egg viability (rather than fecundity), or even affecting the mating success of males.

Unlike *D*. *melanogaster*, the female SWD *Tdc2* null mutants are still able to lay small numbers of viable eggs, and we did not explore the reason for this. We suspect that the insertion of a large gene drive cassette in the *Tdc2* locus might impact neighboring *Tdc1* expression and partially rescue the *Tdc2* phenotype, potentially allowing the issue to be solved with drive design adjustments such as reversing the orientation of some genes within the drive. Another plausible explanation is that the function of *Tdc2* is not fully conserved between these two fly species, implying that SWD *Tdc2* might not be an optimal target gene due to the observed incomplete sterility.

As gene drive elements propagate throughout the population, they can trigger the emergence of resistance alleles due to the end-joining repair, thereby impacting the efficiency of the gene drive. Nonfunctional resistance alleles can impede the spread of the gene drive, especially if they arise from maternally deposited Cas9 and gRNAs. The presence of functional resistance alleles would have an even more significant impact. Comprehensive studies have shown that simultaneous targeting with several gRNAs improves cleavage efficiency and reduces resistance alleles in cage trials [14,15]. To mitigate the formation of functional resistance alleles, we employed three gRNAs to target the *Tdc2* gene, inducing nonfunctional mutations at the desired sites. This design likely reduced the selective pressure for resistance by minimizing the formation rate of functional resistance alleles.

The key characteristic of our gene drive systems is that the phenotype of female sterility can be rescued by feeding of octopamine, which is inexpensive. In certain gene drive systems that aim to induce female infertility by targeting haplosufficient genes, it is impossible to reverse the infertility phenotype in drive homozygous females [6]. Therefore, introduction of wild-type females into the gene drive transgenic population continuously is necessary to maintain the population. In our design, feeding octopamine to female flies can successfully restore their egg-laying defects. This may facilitate large-scale breeding of gene drive flies and contribute to commercial production [9], reducing costs, implying rearing, and potentially even allowing mass-rearing strategies and continuous releases that require reduced drive performance for successful suppression.

## Materials and methods

### Fly strains

The SWD was collected from cherry fields in Tai’an, Shandong Province, China, back in 2012 and has been maintained in the laboratory of Yi Yu (Institute of Plant Protection, Shandong Academy of Agricultural Sciences, Jinan, China) ever since. The *D*. *melanogaster* strains including *w*^*1118*^, *nos*-Cas9(X) (BDSC_54591), *vasa*-Cas9(X) (BDSC_51323), *actin5C*-Cas9(X) (BDSC_54590), *nos*-Cas9(II) (BDSC_78781), *nos*-Cas9(III) (BDSC_78782), and *vasa*-Cas9(X) (BDSC_55821) were all purchased from Bloomington Drosophila Stock Center (Indiana University). We generated SWD and *D*. *melanogaster* mutants by microinjections. All flies were reared in an incubator at 25 ± 1°C, 60% ± 10% humidity with a 12:12 h light/dark cycle. The SWD were fed using sucrose food that was optimized by Bing et al. while *D*. *melanogaster* were fed using conventional cornmeal agar molasses medium [30].

### Target gene

The sequence of *D*. *melanogaster Tyrosine decarboxylase 2* (*DmTdc2*, FlyBase: CG30446), which is located on the right arm of chromosome 2, was found on Flybase (http://flybase.org). The total length of the gene is 5230bp, including 8 exons. Flycrispr Optimal Target Finder (http://flycrispr.molbio.wisc.edu/tools) was used to find possible gRNA target sites. We selected three gRNAs located on the third or fourth coding exon to target *DmTdc2*. For SWD, the protein sequence of *DmTdc2* was used to search the genome sequence of SWD *Tdc2* gene (*DsTdc2*) in the genome database of SWD [19]. According to the annotated information of SWD provided by Chiu et al. [19], the intron and exon parts of *DsTdc2* were analyzed, and then the gRNAs were selected by using Flycrispr Optimal Target Finder.

### Plasmid construction

All plasmids were constructed using homologous recombination technique. Fragments were amplified using KOD One PCR Master Mix (TOYOBO, Japan) and Gibson assembled with ClonExpress MultiS One Step Cloning Kit (Vazyme, China). Plasmids were transformed into Trelief 5α or DB3.1 chemically-competent *E*. *coli* (Tsingke, China), isolated, and sequenced. Full sequences of plasmids and oligos can be found in S2 and S3 Tables. S2 Table lists the Oligonucleotides used in the SWD gene drive system, and S3 Table lists the Oligonucleotides used in the *D*. *melanogaster* gene drive system.

### Microinjection of plasmids

Plasmids were purified by QIAGEN Plasmid Midi Kit (QIAGEN, Germany) and re-sequenced before microinjections. Since the plasmids we constructed expressed both SpCas9 endonuclease and gRNA, a single injection of this exogenous donor plasmid (500ng/μL) into the early embryo of *Drosophila* achieved successful genome editing. We collected embryos of wild type flies (both SWD and *D*. *melanogaster*) for microinjection. The microinjected flies, which were marked as G0 flies, were crossed with wild type males or female flies, and the hatched G1 generation flies were screened and photographed using a stereological microscope MULTIZOOM AZ100M (Nikon, Japan).

### Molecular analysis of mutants

In order to sequence target sites, DNA of flies was extracted by FastPure Cell/Tissue DNA Isolation Mini Kit (Vazyme, Nanjing, China) and was used as a template for a 30 μL PCR reaction. Primers were designed to amplify the sequences that cover the gRNA cut sites. Primers used for either single fly or cage trial deep sequencing analyses can be found in S2 and S3 Tables.

### Fertility assays

5–10 female virgin SWD of different genotypes (WT and gene drive mutants among which were either heterozygotes or homozygotes) were mated with an equal number of WT male SWD. After 5 days of intercross, a single female and a single male were placed in bottles with a grape-sucrose feed on top so that we could change the feed daily and count the eggs. The oviposition quantity of each female fly was counted for 5–7 consecutive days. Yeast was added to the sucrose feed in order to stimulate egg laying. In one group, 25 mg/mL octopamine was mixed with yeast so that flies would ingest octopamine by feeding on yeast.

### Cage trials

Cage trials of SWD proceeded at 25°C with a 12-hour day-night cycle using 250 mL bottles containing standard cornmeal medium and additional sucrose food. Crosses between homozygous male DsTdc2^CRISPR^ carrying the gene drive cassette and WT female flies were carried out to obtain heterozygotes used to seed the initial generation. WT and heterozygous males and virgin females were collected and separately matured for 3–5 days. All cages were seeded at a phenotypic frequency of 25% heterozygotes (15 males, 15 females) and 75% WT (45 males, 45 females). In each generation, flies were allowed to mate and lay eggs for ~120 hours. Then, parents were removed from the cage (Gn), which was kept for 10 days. Subsequent progeny (Gn+1) hatched within five days were all collected to monitor for DsRed and then seed the following generation. This process of random-sampling and passage was continued for ~12 generations. Approximately 10 flies were randomly selected in certain generations for genome typing.

### Maximum likelihood analysis of cages

To quantify drive fitness costs, we modified a maximum likelihood inference framework [31]. The maximum likelihood inference method is implemented in R (version 4.0.3) and is available on GitHub (https://github.com/jchamper/ChamperLab/tree/main/Cas9-Promoters-Homing-Drive). In this model, we assume a single gRNA at the drive allele site and that all resistance alleles are nonfunctional. Each female randomly selects a mate, and produces offspring, the number of which is reduced in drive/wild-type females if they have a fitness cost. Females without a wild-type allele are assumed to be sterile. In the germline, wild-type alleles in drive/wild-type heterozygotes can potentially be converted to either drive or resistance alleles, which are then inherited by offspring. Based on experimental data (pooling for females, weighted pooling for males based on known proportions of ideal and nonideal strains), the drive conversion rate was set to 7.6% for females and 14.6% for males. Based on cage sequencing data that found resistance alleles, but only in one replicate, we set the germline resistance allele formation rate to be half the drive conversion rate in both sexes. Because germline cut rates were low, we assumed no resistance allele formed by maternally deposited Cas9 and gRNA at the early embryo stage.

## Supporting information

S1 Fig Gene structure of the SWD *vasa*. (A) Gene structure of the SWD *vasa* (A). (B) *Dsvasa* promoter used in this study.
(TIF)

S2 Fig Schematic representation of the HDR knock-in construct of Pub-DsRed donor plasmid specifically recognizing *DsTdc2* gene. The plasmid only contains homology arms and the PUb-DsRed gene. (TIF)

S3 Fig Genetic polymorphism analysis. (A) Genetic polymorphism analysis of the homology arm and gRNA targeted sequences in the *DsTdc2* gene in the 26 heterozygous DsTdc2^CRISPR^ males. The same nucleotide sequences are noted in gray. Different nucleotide sequences are noted in green and yellow. Three gRNAs are shown in the Fig, and the left and right homology arms are indicated. Among the 26 flies tested, there were 16 of the first type, 1 of the second type, 4 of the third type, 3 of the fourth type, and 2 of the fifth type. (B) The inheritance rates of five types of flies.
(TIF)

S4 Fig Sequencing and NHEJ alleles analysis of DsTdc2^CRISPR^ SWD. (A) PCR products sequencing chromatograms of wild type (WT) SWD, microinjected G0, G1 heterozygotes DsTdc2^CRISPR^, and G2 resistant mutant at the gRNA1 target site. (B) NHEJ analysis of PCR products from G1 heterozygotes DsTdc2^CRISPR^, and G2 mutants at the gRNA1 and gRNA3 target sites. GRNA Target sites are indicated in green, PAM in yellow, and dashed lines represent the deleted bases. Letter N represent different types of NHEJ. The numbers on the right represent the count of base deletions.
(TIF)

S5 Fig The number of embryos in each ovary of 20-day-old WT and DsTdc2^CRISPR^ SWD. Data was analyzed using unpaired two-tailed *t*-test, n1 = n2 = 8, *P*<0.0001.
(TIF)

S6 Fig Sequencing and NHEJ alleles analysis of mutants in cage trail. (A) PCR products sequencing chromatograms of WT and resistant mutant in cage 2 (Generation 10) at the gRNA1 and gRNA3 target site. (B) NHEJ analysis of PCR products from resistant mutant in cage 2 (Generation 10) at the gRNA1 and gRNA3 target sites.
(TIF)

S7 Fig Sequencing and NHEJ alleles analysis of mutants mediated by Act5C-Cas9(II). (A) PCR products sequencing chromatograms of WT, heterozygous DmTdc2^Actin5C^, and G2 resistant mutant at the gRNA3 target site. (B) Statistics of resistance alleles in G2 progeny mediated by endogenous Act5C-Cas9(II). As for two DmTdc2^Actin5C^ G1 males, 77 and 61 of their G2 offspring were randomly sampled and analyzed. And for two DmTdc2^Actin5C^ G1 females, 34 of their G2 offspring were tested.
(TIF)

S8 Fig Sequencing and NHEJ alleles analysis of mutants mediated by *nos*-Cas9(II) and *nos*-Cas9(III). (A) PCR products sequencing chromatograms of wild type *w*^*1118*^, heterozygous master G1 mediated by *nos*-Cas9(III) and G2 resistant mutant at the gRNA3 target site. (B) NHEJ analysis of PCR products from G2 resistant mutant mediated by *nos*-Cas9(III) at three gRNA target sites. Base insertions are represented by green uppercase letters, while base substitutions are indicated in red. The numbers on the right represent the count of base deletions, insertions or substitutions. (C) Statistics of resistance alleles in G2 progeny mediated by *nos*-Cas9(II) and *nos*-Cas9(III). As for a G1 male mediated by *nos*-Cas9(II), 75 of their G2 offspring were randomly sampled and analyzed. And for two G1 females mediated by *nos*-Cas9(III), 38 and 37 of their G2 offspring were tested.
(TIF)

S9 Fig Sequencing and NHEJ alleles analysis of mutants mediated by *vasa*-Cas9(X). (A) Heterozygous G1 master female mediated by *vasa*-Cas9(X) were sterile due to egg retention. (B) PCR product sequencing chromatograms of WT, heterozygous master G1 and G2 resistant mutant at the gRNA3 target site. (C) NHEJ analysis of PCR products from G2 resistant mutant of G1 master female mediated by *vasa*-Cas9(X) at three gRNA target sites. (D) Statistics of resistance alleles in G2 progeny (from two different males, left and right pie charts) mediated by *vasa*-Cas9(X). Specifically, 65 and 59 of their G2 progeny were sampled and analyzed.
(TIF)

S1 Table Maximum likelihood estimates of female drive heterozygote fitness. (XLSX)

S2 Table Oligonucleotides used in *Drosophila suzukii* gene drive system. (XLSX)

S3 Table Oligonucleotides used in *Drosophila melanogaster* gene drive system. (XLSX)

S1 Data Quantitative data used for graphs presented in Figs 2–4 and S4. (XLSX)
