# Supplementary figures and images for: A small-molecule approach to restore female sterility phenotype targeted by a homing suppression gene drive in the fruit pest Drosophila suzukii
Source: PLoS Genet. 2024 Apr 5;20(4):e1011226. doi: 10.1371/journal.pgen.1011226 (PMC11023630; doi:10.1371/journal.pgen.1011226)

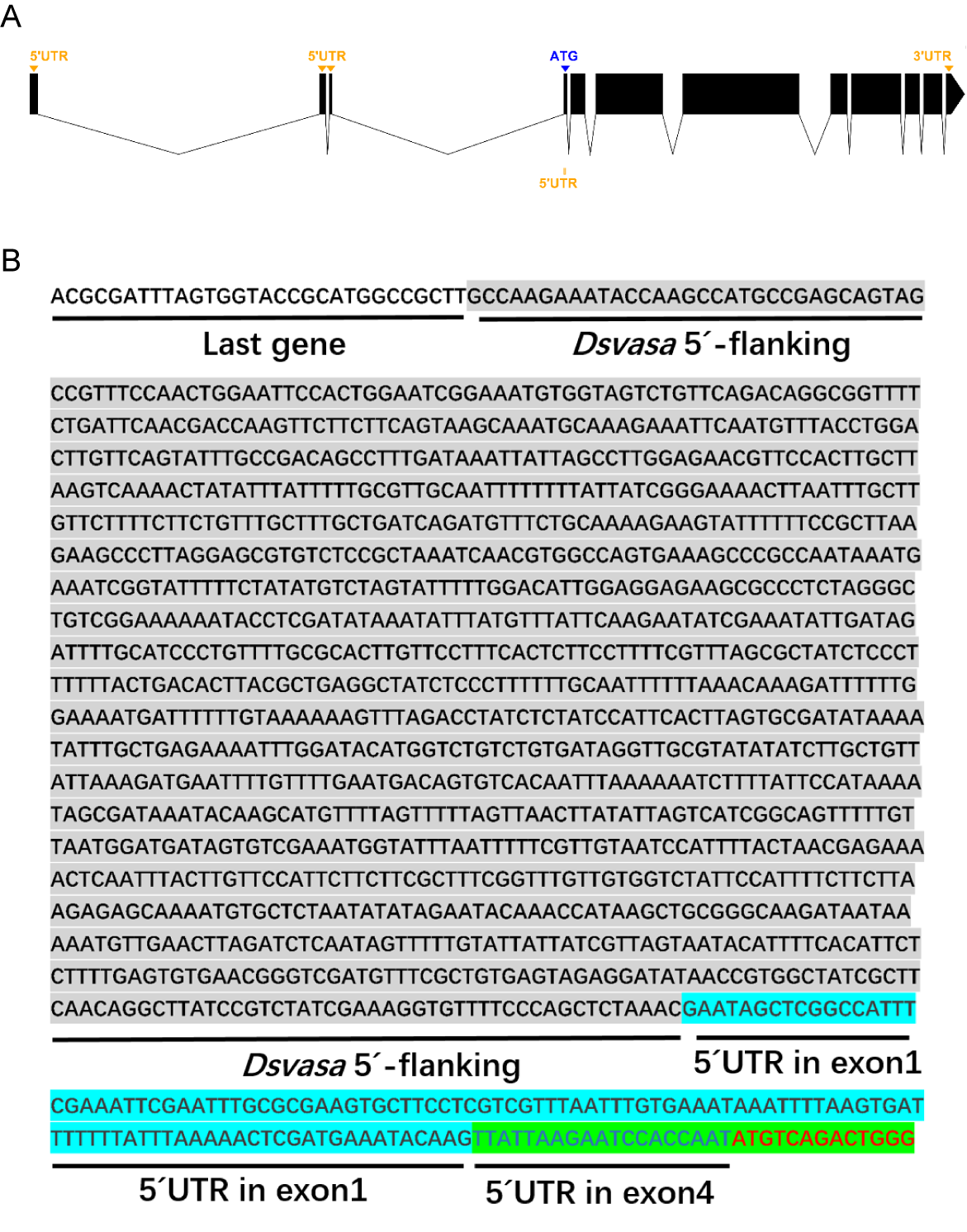

Supplement: S1 Fig — (A) Gene structure of the SWD vasa (A). (B) Dsvasa promoter used in this study. (TIF) [file pgen.1011226.s001.tif]

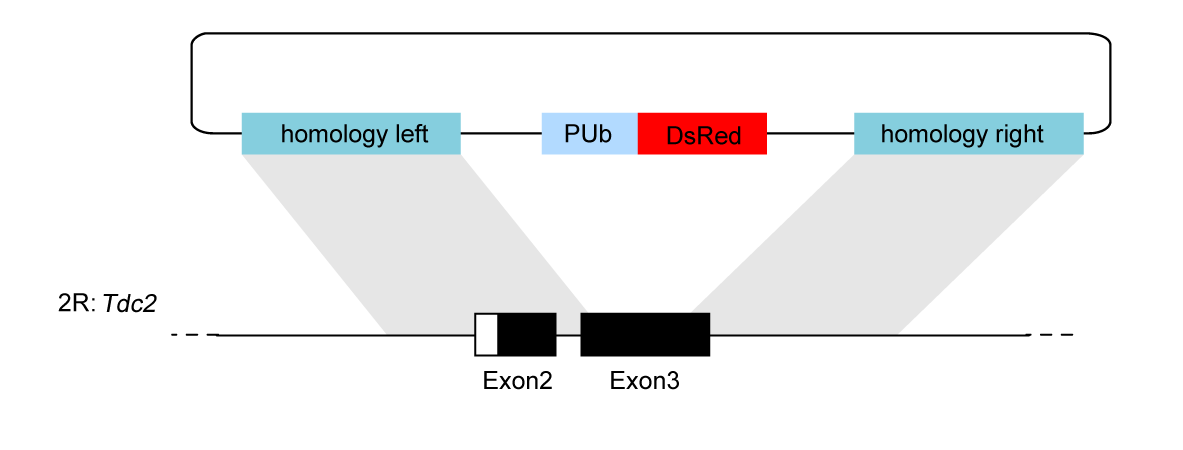

Supplement: S2 Fig — (TIF) [file pgen.1011226.s002.tif]

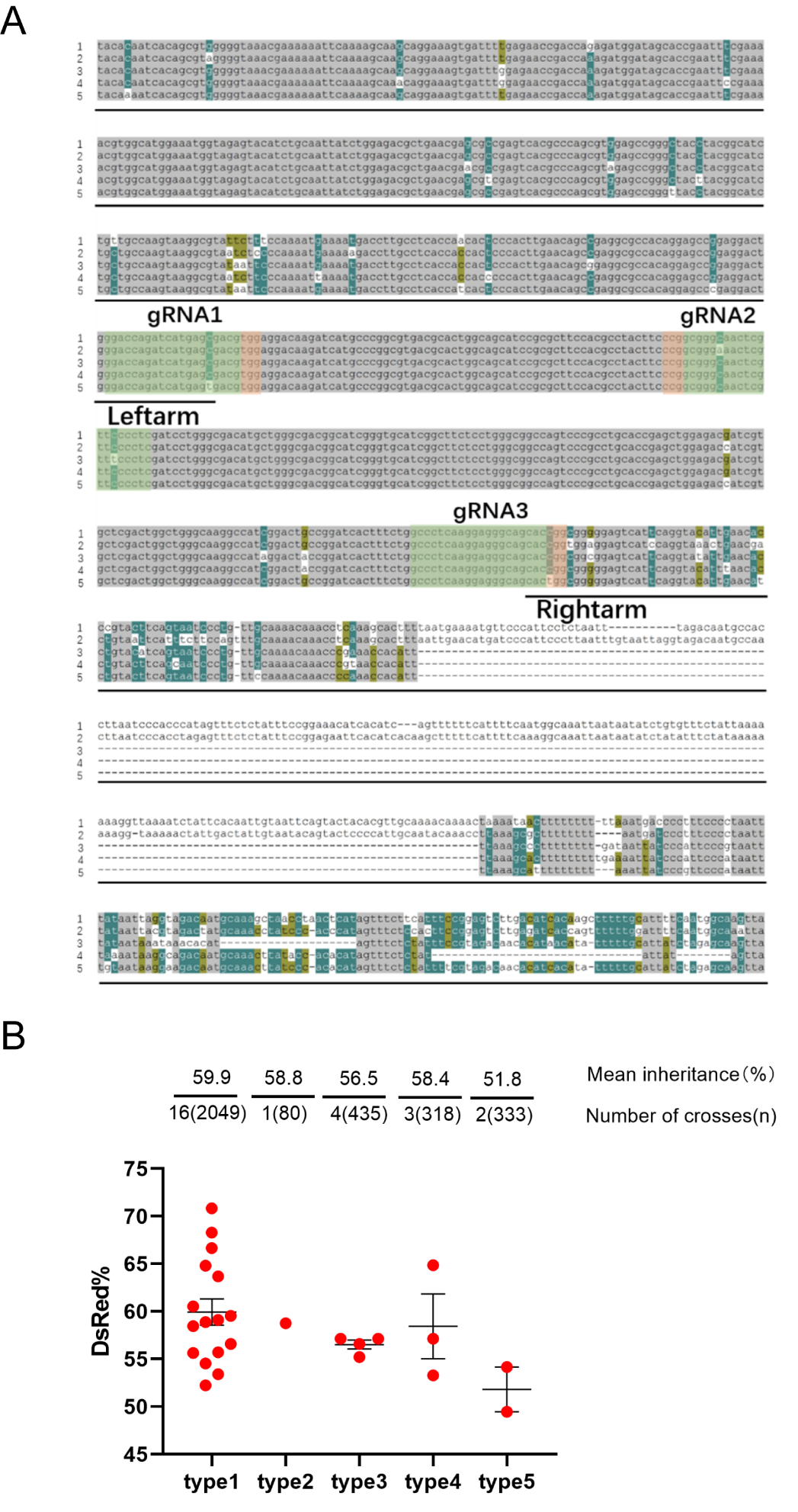

Supplement: S3 Fig — (A) Genetic polymorphism analysis of the homology arm and gRNA targeted sequences in the DsTdc2 gene in the 26 heterozygous DsTdc2CRISPR males. The same nucleotide sequences are noted in gray. Different nucleotide sequences are noted in green and yellow. Three gRNAs are shown in the Fig, and the left and right homology arms are indicated. Among the 26 flies tested, there were 16 of the first type, 1 of the second type, 4 of the third type, 3 of the fourth type, and 2 of the fifth type. (B) The inheritance rates of five types of flies. (TIF) [file pgen.1011226.s003.tif]

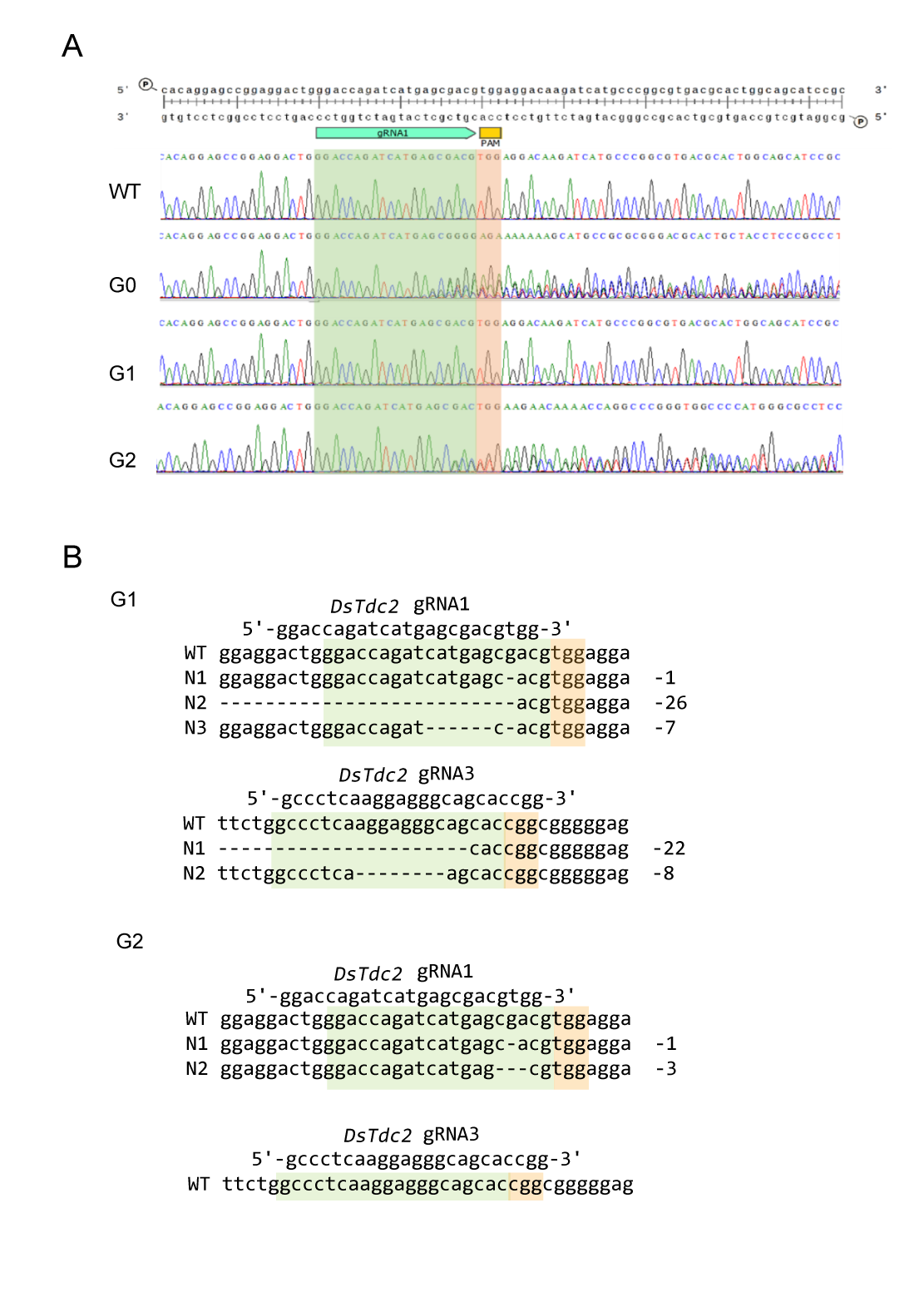

Supplement: S4 Fig — (A) PCR products sequencing chromatograms of wild type (WT) SWD, microinjected G0, G1 heterozygotes DsTdc2CRISPR, and G2 resistant mutant at the gRNA1 target site. (B) NHEJ analysis of PCR products from G1 heterozygotes DsTdc2CRISPR, and G2 mutants at the gRNA1 and gRNA3 target sites. GRNA Target sites are indicated in green, PAM in yellow, and dashed lines represent the deleted bases. Letter N represent different types of NHEJ. The numbers on the right represent the count of base deletions. (TIF) [file pgen.1011226.s004.tif]

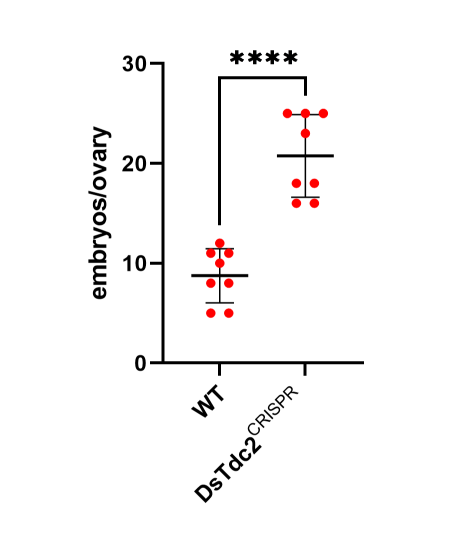

Supplement: S5 Fig — Data was analyzed using unpaired two-tailed t-test, n1 = n2 = 8, P<0.0001. (TIF) [file pgen.1011226.s005.tif]

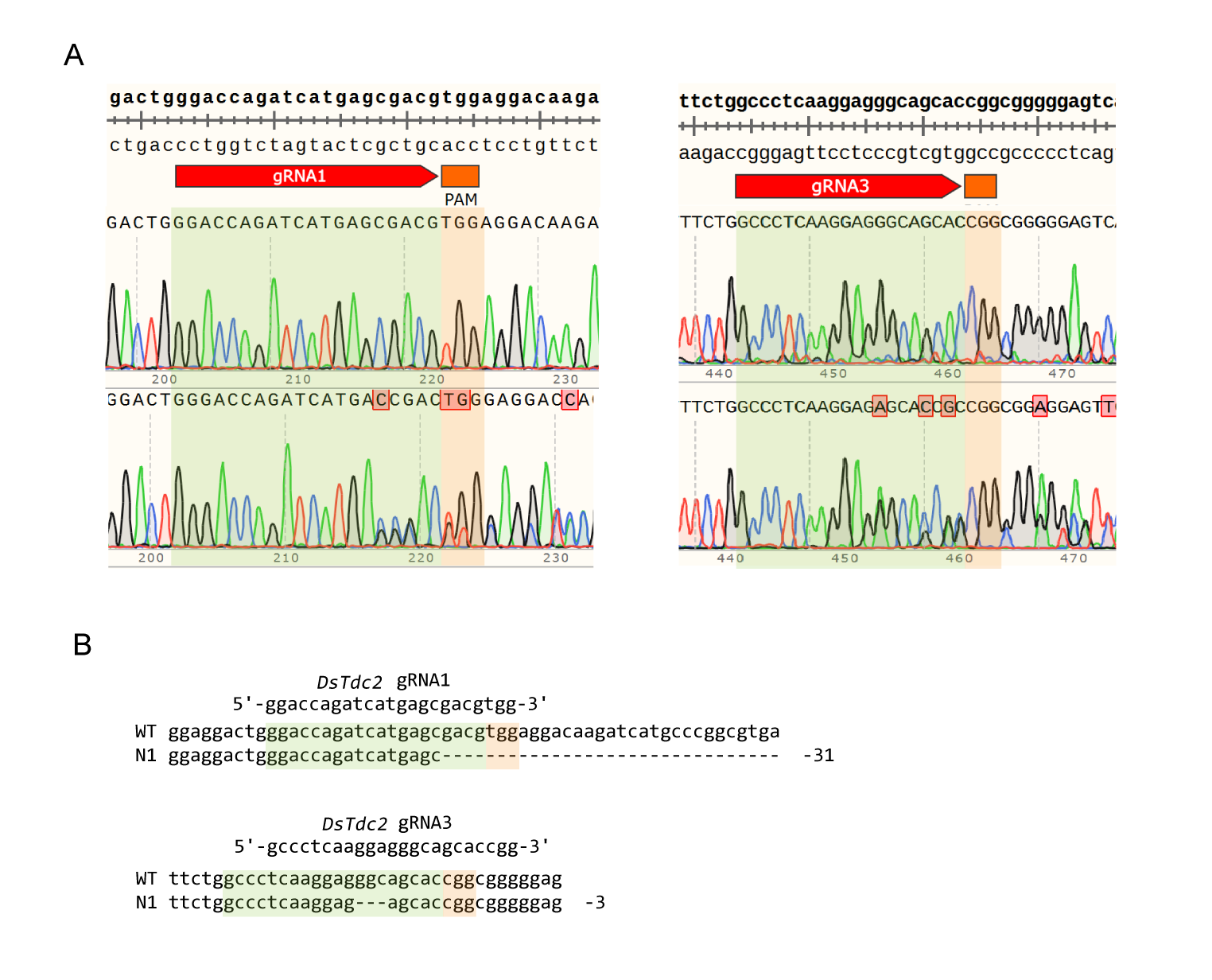

Supplement: S6 Fig — (A) PCR products sequencing chromatograms of WT and resistant mutant in cage 2 (Generation 10) at the gRNA1 and gRNA3 target site. (B) NHEJ analysis of PCR products from resistant mutant in cage 2 (Generation 10) at the gRNA1 and gRNA3 target sites. (TIF) [file pgen.1011226.s006.tif]

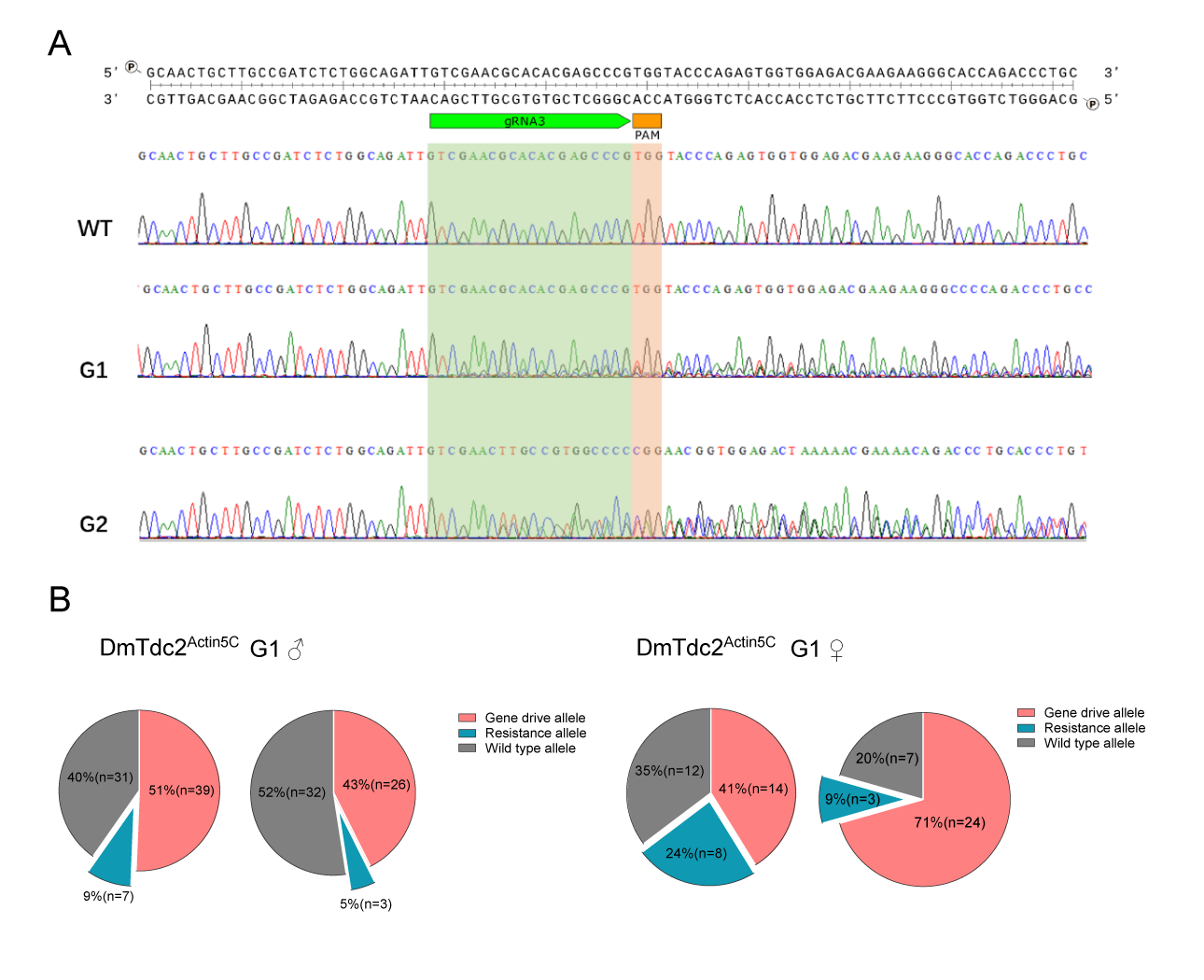

Supplement: S7 Fig — (A) PCR products sequencing chromatograms of WT, heterozygous DmTdc2Actin5C, and G2 resistant mutant at the gRNA3 target site. (B) Statistics of resistance alleles in G2 progeny mediated by endogenous Act5C-Cas9(II). As for two DmTdc2Actin5C G1 males, 77 and 61 of their G2 offspring were randomly sampled and analyzed. And for two DmTdc2Actin5C G1 females, 34 of their G2 offspring were tested. (TIF) [file pgen.1011226.s007.tif]

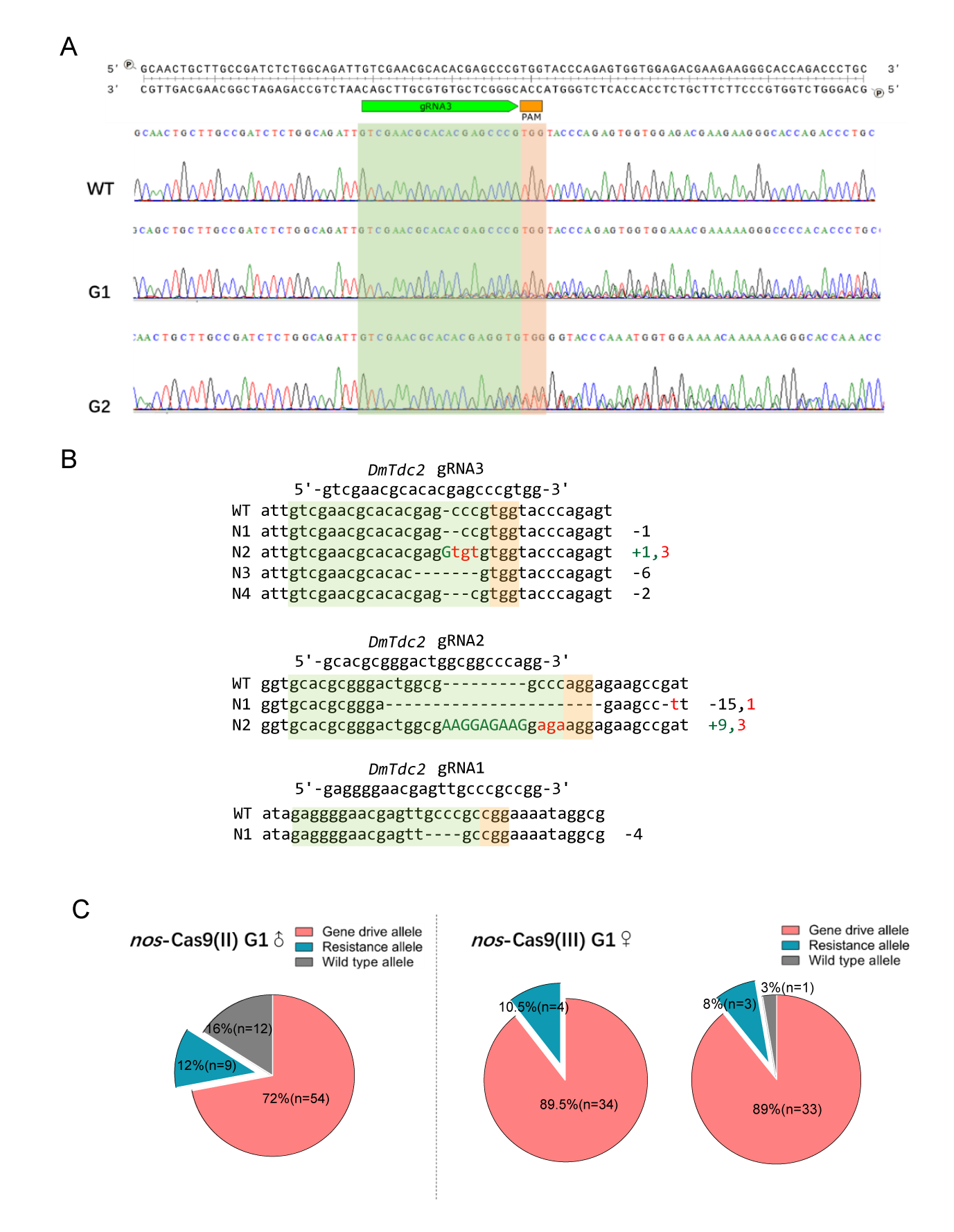

Supplement: S8 Fig — (A) PCR products sequencing chromatograms of wild type w1118, heterozygous master G1 mediated by nos-Cas9(III) and G2 resistant mutant at the gRNA3 target site. (B) NHEJ analysis of PCR products from G2 resistant mutant mediated by nos-Cas9(III) at three gRNA target sites. Base insertions are represented by green uppercase letters, while base substitutions are indicated in red. The numbers on the right represent the count of base deletions, insertions or substitutions. (C) Statistics of resistance alleles in G2 progeny mediated by nos-Cas9(II) and nos-Cas9(III). As for a G1 male mediated by nos-Cas9(II), 75 of their G2 offspring were randomly sampled and analyzed. And for two G1 females mediated by nos-Cas9(III), 38 and 37 of their G2 offspring were tested. (TIF) [file pgen.1011226.s008.tif]

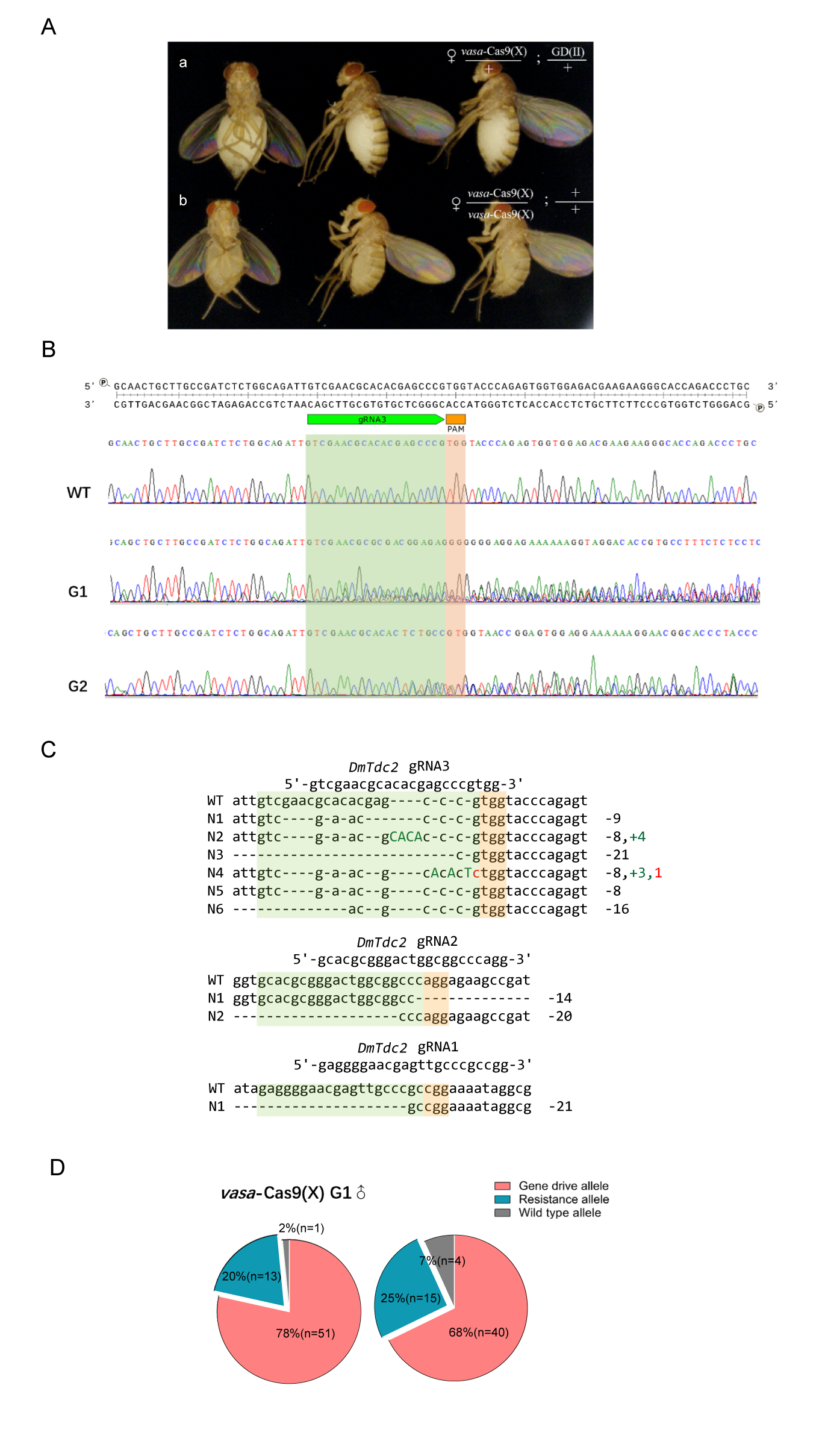

Supplement: S9 Fig — (A) Heterozygous G1 master female mediated by vasa-Cas9(X) were sterile due to egg retention. (B) PCR product sequencing chromatograms of WT, heterozygous master G1 and G2 resistant mutant at the gRNA3 target site. (C) NHEJ analysis of PCR products from G2 resistant mutant of G1 master female mediated by vasa-Cas9(X) at three gRNA target sites. (D) Statistics of resistance alleles in G2 progeny (from two different males, left and right pie charts) mediated by vasa-Cas9(X). Specifically, 65 and 59 of their G2 progeny were sampled and analyzed. (TIF) [file pgen.1011226.s009.tif]
